# COVID-19 and Its Cardiovascular Effects: Risk Factors, Prevention and Management

**DOI:** 10.3390/jcm12134457

**Published:** 2023-07-03

**Authors:** Celestino Sardu, Raffele Marfella

**Affiliations:** Department of Advanced Medical and Surgical Sciences, University of Campania, “Luigi Vanvitelli”, 80126 Naples, Italy; raffaele.marfella@unicampania.it

Coronavirus disease 2019 (COVID-19) is caused by a positive-stranded, single-stranded RNA virus, which is a member of the Sarbecovirus subgenus (beta-CoV lineage B) [1,2].

This RNA virus is responsible for severe acute respiratory syndrome (SARS-CoV-2), and it was first isolated in December 2019 in patients from the city of Wuhan [1,2]. 

COVID-19 manifests in a range of different levels of severity. SARS-CoV-2-positive individuals could remain asymptomatic (ASAP), or they could display a more severe progression of the clinical form, thus requiring intubation, mechanical ventilation or intensive care unit (ICU) admission; the virus can also cause death [1,2]. Notably, a higher percentage of patients diagnosed with severe forms of COVID-19 have type two diabetes mellitus (T2DM) [3,4], hypertension and cardiovascular disease (CVD) [5,6]. These patients could experience worse prognoses and higher rates of death [1,2,3,4,5,6]. 

Intriguingly, SARS-CoV-2 infection could lead to over-inflammation in these patients, with endothelial dysfunction and a hyper-coagulative status [3,4,5,6,7]. The unbalanced activation of these molecular and cellular pathways could be increased by triggering factors. 

In this context, it has been found that hyperglycemia at hospital admission could trigger a more severe form of COVID-19, and it has been shown to be a risk factor for a worse prognosis in patients with COVID-19 [8]. Notably, while about 7% of patients hospitalized due to COVID-19 have T2DM, hyperglycemia has been observed in up to 50% of patients hospitalized due to COVID-19 [8]. 

Notably, hyperglycemia increases the serum concentrations of pro-inflammatory cytokines, thus affecting the response of the innate immune system to SARS-CoV-2 [8].

These negative effects on the inflammatory burden and immune system are associated with poorer outcomes in patients with mild, moderate and severe COVID-19 [8]. 

Conversely, as seen in patients with T2DM, hyperglycemia could alter the expression of Angiotensin-Converting Enzyme 2 (ACE2) pathways at the cardiomyocyte level [9].

The ACE2 receptor and its pathways represent the door through which SARS-CoV-2 enters and replicates into the cardiomyocytes [9]. In this context, hyperglycemia could affect the avidity of intramyocardial binding between ACE2 and SARS-CoV-2, and this could cause higher rates of myocarditis, cardiac complications, heart failure (HF) and death [9].

From this evidence, it is important to define and identify the altered mechanisms which are responsible for worse prognoses in higher-risk populations. Firstly, the early diagnosis of SARS-CoV-2 infection and COVID-19 would be beneficial. Secondly, it is necessary to use the best management and therapeutic approaches for patients with COVID-19. In this context, these novelties in the diagnostic and therapeutic field could be relevant to controlling the cardiovascular risk, ameliorating clinical outcomes and reducing the mortality rate in SARS-CoV-2-positive and COVID-19 patients. In this Special Issue, the authors mainly focused on cardiovascular effects in patients with COVID-19. The authors published interesting and innovative data regarding diagnostic and therapeutic approaches related to molecular, cellular and epigenetic biomarkers of cardiovascular disease in patients with COVID-19. The authors particularly focused on the development of treatment strategies to control and/or revert these clinical and pathological adaptive conditions [10,11,12,13,14,15].

**COVID-19, over-inflammation and CVD**

In this setting, the authors investigated the negative association between inflammatory markers, specifically Interleukin-32 (IL-32) and Interleukin-34 (IL-34), with CVD and short-term mortality in patients with COVID-19 [10]. The authors collected the serum levels of IL-32 and IL-34 from patients on admission, which were tested to determine their association with CVD and short-term mortality in 245 patients with COVID-19 [10]. Notably, in this study, 37 patients (15.1%) reached the primary endpoint of 28-day mortality [10]. Despite this, IL-32 and IL-34 did not show any associations with CVD or 28-day mortality in the context of COVID-19, while the patients with multiple CVD had a significantly increased risk of short-term mortality [10]. Therefore, this important research shows that CVD could represent a relevant risk factor for a worse prognosis in COVID-19 patients, as well as the different stages of inflammation [3,4,5,6,10]. 

**Preventive and therapeutic approaches in cohorts of COVID-19 patients with CVD**

To date, authors have focused on developing preventive and therapeutic approaches in cohorts of COVID-19 patients with CVD. Thus, the best treatment of CVD risk factors and parallel factors must be promoted to continue therapies for the treatment of CVD in patients with COVID-19. In line with this observation, other authors analyzed data from a cohort of patients divided into two groups of patients: statins users and non-users of lipid-lowering therapies (non-LLT users) [11]. In these cohorts, the authors evaluated the relationship between exposure to statins and the risk of requiring mechanical ventilation (MV), ICU access and death at 30 days of follow-up [11]. They found a decreased risk of mortality at 30 days (HR: 0.39; 95% CI: 0.18–0.85) in statins users compared with non-LLT users [11]. Moreover, the authors suggested that the utilization of LLT to reduce lipid levels and atherosclerosis progression might induce the best clinical outcomes in patients with COVID-19. Parallel to LLT (and other anti-CVD treatments), the authors showed that patients receiving hyperbaric oxygen therapy for post-COVID-19 syndrome had the best clinical outcomes at long-term follow-up post COVID-19 [12]. They showed that the use of 15 compression sessions was temporarily associated with a noticeable improvement in health and performance parameters and in certain blood gas parameters [12].

**Diagnostic, monitoring and therapeutic biomarkers and clinical outcomes in patients with severe COVID-19**

Valuable and reproducible diagnostic, monitoring and therapeutic biomarkers are critical for improving clinical outcomes in patients with severe COVID-19. In this setting, recently, authors evaluated catestatin in patients with severe COVID-19 hospitalized in the ICU [12]. Catestatin is a pleiotropic peptide which has multiple immunomodulatory effects [13]. In a multivariate logistic regression analysis, catestatin predicted COVID-19 survival [13]. Conversely, a few authors found that patients with COVID-19 may manifest thrombocytopenia, and some may succumb to infection due to coagulopathy [2,14]. Notably, COVID-19 survivors showed an average platelet value at entry to the emergency department of 220.1 ± 81.4, while that of those who died was 206.4 ± 87.7 [14]. Furthermore, SARS-CoV-2 infection may induce thrombocytopenia, and the reduction in platelet counts could correlate with the main blood gas parameters and with the clinical outcome [14]. Therefore, authors have suggested that the platelet count could be an important prognostic factor to evaluate and stratify patients with COVID-19 [14].

**Vaccination strategy in patients with COVID-19**

Last but not least, it has been necessary for authors to examine COVID-19 populations treated with a vaccination strategy and the negative impact caused by altered glucose homeostasis and hyperglycemia in these cohorts of patients [15,16,17]. Acute hyperglycemia at hospital admission is a risk factor for worse COVID-19 prognosis in patients with and in those without T2DM [15,16,17]. Acute and chronic glycemic control could be identified as significant determinants of vaccination efficacy, disease severity and mortality rate in patients with COVID-19 [15,16,17]. 

Hyperglycemia might affect the prognosis of patients with COVID-19 through multiple mechanisms [15,16,17]. In this setting, authors have considered the induction of the glycation and oligomerization of ACE2 (the main receptor of SARS-CoV-2), the increased expression of the serine protease TMPRSS2 (responsible for S protein priming) and the impairment of the function of innate and adaptive immunity [15,16,17]. Furthermore, the treatment of acute hyperglycemia through insulin infusion could improve clinical outcomes in patients with COVID-19 [8,9]. Conversely, the achievement of the best chronic glycemic control could positively affect the immune response following vaccination. Looking at these data, authors would encourage more researchers from the scientific field to consider consequent and possible applications in the clinical setting with regard to the prevention, diagnosis and treatment of COVID-19 in the overall population, particularly in those at high risk of worse cardiovascular outcomes. 

As the Guest Editor of the current Special Issue, I would like to thank all of the authors for their valuable contributions, the reviewers for their time in critically reviewing the papers and making important comments and the *JCM* editorial team for their collective support and assistance. This Special Issue includes several interesting papers that will help clinicians in decision making and treatment choices. This Special Issue addresses new aspects of COVID-19 and its severity, particularly in high-risk patients with CVD. Therefore, from the current Special Issue and the articles published, the authors have presented a few key points that help elucidate the implications and future directions of research in the field of COVID-19. For example, the diagnosis of multiple CVD and the over-expression of inflammatory cytokines (IL-32 and IL-34) could increase short-term mortality rates [10]. Therefore, preventive LLT therapies could decrease mortality risk at 30 days via the best-known control of CVD [11]. Notably, clinical complications could persist for several months after initial recovery from COVID-19. In this context, hyperbaric oxygen therapy could ameliorate clinical outcomes at long-term follow-up post COVID-19 [12]. Conversely, new diagnostic and prognostic biomarkers could help increase survival rates in patients with COVID-19. In this setting, authors evaluated the serum catestatin in patients with severe COVID-19 hospitalized in the ICU [13] and the platelet counts in survivors of COVID-19 [14]. Intriguingly, catestatin levels could predict COVID-19 survival [13], and the survivors of COVID-19 had lower average platelet counts at entry to the emergency department [14]. Last but not least, the vaccination strategy could reduce the disease severity and mortality rate in patients with COVID-19 [15,16,17]. Despite this, the vaccination strategy and its efficacy could be reduced by altered glucose homeostasis and hyperglycemia in these cohorts of patients [15,16,17].
